# Schwannoma of Seminal Vesicle: A Case Report

**DOI:** 10.7759/cureus.32986

**Published:** 2022-12-27

**Authors:** Diogo Pereira, Gabriel Costa, Raquel Catarino, Tiago Correia, Rui Prisco

**Affiliations:** 1 Urology, Hospital Pedro Hispano, Matosinhos, PRT

**Keywords:** rare tumors, minimaly invasive surgery, lower urinary tract synptoms, seminal vesicle, peripheral schwannoma

## Abstract

Seminal vesicle neoplasms are extremely rare. Schwannoma is a benign tumor of the peripherical nerve sheath composed of Schwann cells. Most of these tumors are silent and become symptomatic with compression of adjacent organs and nerves. We present a case of a 72-year-old man who presented with a several months history of predominant storage lower urinary tract symptoms and painful ejaculation. Prostate-specific antigen (PSA) was within normal ranges, and imaging documented a retrovesical nodular lesion adjacent to the right seminal vesicle with 5 cm in width. We successfully performed a robotic-assisted laparoscopic surgery to excise the lesion. Anatomopathological analysis revealed a schwannoma.

## Introduction

Neoplasms of the seminal vesicles are a relatively rare entity. Within these, benign neoplasms are the most frequent and include fibromas, cystadenomas, leiomyomas, schwannomas, and papillary adenomas. Otherwise, primary malignant neoplasms include adenocarcinoma of the seminal vesicle, sarcomas (leiomyosarcoma, rhabdomyosarcoma, angiosarcoma, Mullerian adenosarcoma-like tumor, cystosarcoma phylloides), seminoma, squamous cell carcinoma, and extra-gastrointestinal stromal tumors [1]. These are extremely rare, and only sporadic cases are described in the literature. Schwannomas are the most common benign tumors of the peripheral nerves. The aim of this article is to describe a clinical case of a patient with a seminal vesicle schwannoma.

## Case presentation

This is a case report of a 72-year-old man with well-controlled arterial hypertension and no past surgical or family history. 

He was referred to a urology appointment because of predominant storage lower urinary tract symptoms, namely pollakiuria, urgency and straining, and painful ejaculation with several months of evolution. The International Prostate Symptom Score (IPSS) was 13, and the patient was mostly dissatisfied with his urinary condition. A digital rectal examination revealed a prostate with a fibro-elastic consistency, well-defined edges, and an apparent mass in the region of the right seminal vesicle detached from the prostate. The PSA was within the normal range (2.5 ng/mL), with normal renal function and urinalysis. Uroflowmetry demonstrated an arc-shaped curve with a Qmax of 13.5 mL/seg. 

**Figure 1:**
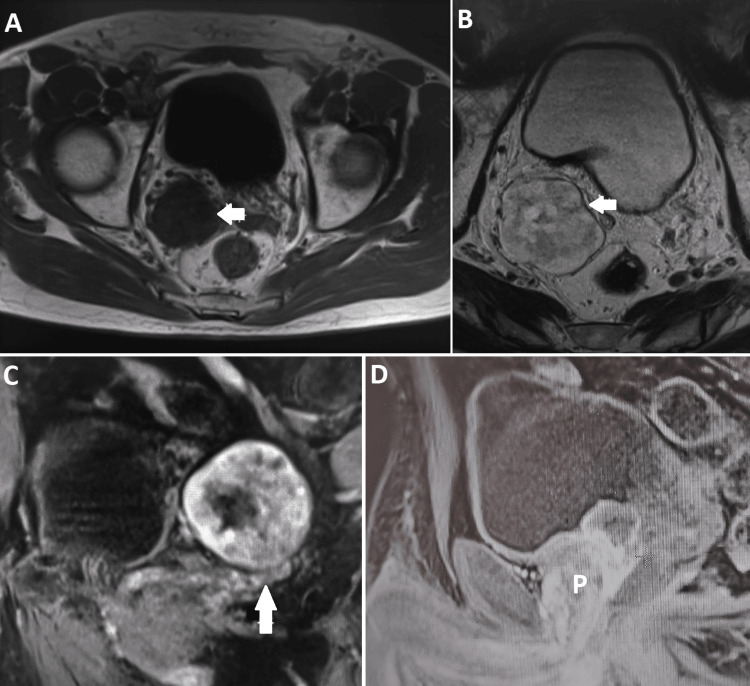
MRI showing nodular and polylobulated mass postero-lateral to the bladder (arrows), in close contact with the right adrenal vesicle. A: T1-weighted MRI showed hyposignal with avid contrast uptake, a small non-enhancing central zone, and restriction to water diffusion in several zones. B: T2-weighted MRI presented heterogeneous hypersignal. C: Sagittal plane. D: Saggital plane: relation with prostate (P).

Transrectal ultrasound showed a 5 cm wide nodular image adjacent to the right seminal vesicle. The prostate was 54 cc. He subsequently underwent a pelvic MRI, which showed a well-delineated nodular and polylobulated mass measuring 54 x 49 x 53 mm, posterior and lateral to the bladder, in close contact with the right seminal vesicle (Figure 1). There were no other findings of significance in the bladder, prostate, or rectum in the MRI.

The patient was counseled regarding the risks, benefits, and alternatives to surgical resection. A successful transperitoneal robotic-assisted excision of the mass was performed. Under general anesthesia, the patient was placed in a dorsal lithotomy position. Ports were placed in the standard W configuration with an assistant port in the right lower quadrant, like other pelvic surgeries. We started by identifying the right ureter at its intersection with the iliac vessels. This was dissected free and suspended with a vessel loop to facilitate touchless manipulation. It was mobilized distally towards the ureterovesical junction, where the tumor of the seminal vesicle was found. The retrovesical mass was not adherent to any surrounding structure and was carefully dissected. The seminal vesicle and the right vas deferens were identified. The mass was removed with a segment of the right seminal vesicle attached through the supraumbilical incision. The total operative time was 90 minutes without any adverse events. The postoperative period was well tolerated, and the patient was discharged two days later.

**Figure 2:**
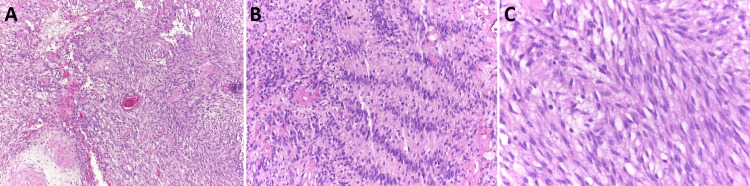
Anatomopathological result. Fusocellular neoplasia compatible with schwannoma. A: Antoni B pattern with a looser stroma, fewer cells, and myxoid change; H&E staining, original magnification x10. B: More cellular "Antoni A" pattern with palisading nuclei surrounding pink areas; H&E staining, original magnification x40. C: Interlacing fascicles of spindle cells; H&E staining, original magnification x100.

The histopathological and immunohistochemical analysis showed a fusocellular neoplasia composed of cells with elongated nuclei expressing S100 protein. In contrast, CD34 and NF markers were negative and compatible with schwannoma (Figure 2). The segment of the seminal vesicle present in the sample was free of neoplasia, and there were no malignancy criteria.

After surgery, the patient experienced an improvement in his storage urinary symptoms with an IPSS of 5, no complaints of erectile or ejaculatory dysfunction, and no signs of recurrence after one year of follow-up.

## Discussion

Schwannomas are the most common benign tumors of peripheral nerves [2]. They are encapsulated tumors originating in Schwann cells. Histologically, they are composed of alternating areas of dense cellularity termed Antoni-A regions and areas of myxoid matrix termed Antoni-B regions. Immunohistochemical staining is typically positive for S-100 and negative for CD34, epithelial markers, and smooth muscle-specific actin. [3]. NF is usually positive in other diseases like neuroblastoma or Medulloblastoma. Malignant progression is rare; however, a pathological variant (melanotic schwannoma) has been described in which malignant transformation can occur [3, 4]. Most schwannomas are single and sporadic, affecting individuals of all ages but with a peak occurring between 20 and 50 years, with similar prevalence in both sexes [5]. When these are multiple, they are usually associated with familial syndromes such as neurofibromatosis type 2, Schwannomatosis, and Carney Complex [6]. They can be found more commonly on the head and neck but on any other body part, namely, limbs, chest, abdomen, retroperitoneum, and pelvic cavity. Schwannoma of the seminal vesicle is extremely rare, with only twelve cases described to our knowledge. The first case was described by Iqbal N et al. [7] in 2002. They are usually asymptomatic and diagnosed incidentally or when the tumor becomes large enough to compress adjacent structures.

The etiological study usually includes an imaging exam (CT scan and/or MRI) to characterize the location, size, and extent of the tumor. Most cases reported to date included a transrectal biopsy for histological characterization. He R et al. [8] described a case of a cystic schwannoma, considering that a biopsy would not be appropriate for this situation. Autieri D et al. [9] described a case of a transrectal biopsy of a schwannoma of the seminal vesicle complicated with an abscess that needed urgent drainage. In the case we present, we think that the previous histological diagnosis would not have changed the decision to perform the excision, as the retrovesical mass was symptomatic. As we considered the possibility of an extra-gastrointestinal stromal tumor as a differential diagnosis, the biopsy could then have caused complications such as a rupture of the tumor, hemorrhage, or tumor spread [10, 11]. The mainstay of treatment for seminal vesicle schwannomas remains surgical excision, particularly when symptomatic. Otherwise, clinical surveillance is a viable option. Surgery can be performed by open procedure or laparoscopy. To our knowledge, this case is the second to describe a robot-assisted approach, with the inherent advantages of this minimally invasive technique: decreased surgical risk, decreased hospital stay, and improved cosmetic outcomes compared to an open surgical procedure. However, the excision must be complete, and in case of incomplete excision, schwannomas may recur.

## Conclusions

Seminal vesicle tumors are rare, and their clinical diagnosis and surgical approach can be challenging. Imaging studies play an essential role in the diagnosis. The presence of a symptomatic seminal mass should prompt surgery. In our opinion, a robot-assisted approach is an excellent option for this pathology.
